# Exploring Bacterial Interactions Under the Stress Gradient Hypothesis in Response to Selenium Stress

**DOI:** 10.1111/1758-2229.70191

**Published:** 2025-09-05

**Authors:** Kristian J. Harris, Alison E. Bennett

**Affiliations:** ^1^ Department of Evolution, Ecology, and Organismal Biology The Ohio State University Columbus Ohio USA

**Keywords:** bacterial facilitation, bioremediation, microbial interactions, oxidative stress, selenium stress, stress gradient hypothesis

## Abstract

The Stress Gradient Hypothesis (SGH) predicts that interspecific interactions shift from competition under low stress to facilitation under high stress. Historically, this framework has been extensively studied in plants, but its application to microbial communities remains underexplored. Here, we review literature to examine bacterial interactions under heavy metal stress, using selenium (Se) stress as a model for heavy metal‐induced environmental pressures. Se, a naturally occurring and anthropogenic metalloid contaminant, provides oxidative stress on bacteria, which will modify competitive and facilitative behaviours under the SGH framework. At low Se concentrations, bacterial interactions are predominantly competitive, driven by resource competition and antimicrobial strategies. However, as Se stress increases, we predict facilitative interactions to increase, including detoxification mechanisms that reduce toxicity for Se intolerant species. We discuss methodologies to measure bacterial competition and facilitation, propose experimental approaches to identify the transition between these interaction modes, and explore the implications of species richness in microbial stress resilience. Understanding these interactions provides insights into microbial ecology, biogeochemical cycling and potential applications in bioremediation.

## Graphical abstract

Under the model of the stress gradient hypothesis, as selenium (Se) stress increases, bacterial interactions shift from net competitive (low stress) to net facilitative/helpful interactions (high stress). This allows Se susceptible species to benefit from detoxification mechanisms provided by Se detoxifying bacteria which remove toxins from the environment.

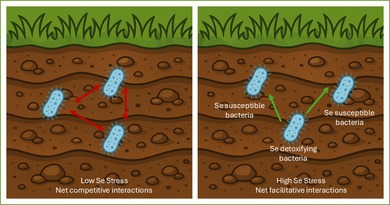

## 1 Introduction

It is well documented that plant species interactions shift in the presence of abiotic stress (He et al. 2013). The stress gradient hypothesis (SGH), as generalised by Maestre et al. (2009) to include non‐plant species, describes how abiotic stress shapes communities through a shift from competitive to facilitative interactions. The SGH initially focused on plants to describe direct, non‐trophic, positive interactions (Bertness and Callaway 1994), but more recent studies have included other organisms such as invertebrates, fungi and bacteria (Hammarlund and Harcombe 2019; Van Alstyne et al. 2020). Although the main focus of the SGH is to determine the effects of abiotic stressors, stress gradients of biotic factors may influence the support for the SGH observed in a study (Jorna et al. 2024). This is highlighted in (Hernandez et al. 2021) where species interactions and environmental filtering relate to community outcomes under stress, and in (Mandakovic et al. 2023) where altitude gradient effects are modulated by plant interactions and environmental stress. A recent meta‐analysis suggests that bacteria likely behave as predicted by the SGH (Adams et al. 2022); however, only 11.1% of papers (185 papers) analysed bacteria. Although Adams' work provides an important repository for interspecies interactions, their literature search only covers work up to 2019 and did not specifically consider experiments under the SGH umbrella but considered studies that could theoretically be considered under the SGH framework. Thus, more work is needed to confirm this result.

We expect that in low stress, resource‐sufficient environments, bacteria compete for space and resources (Ghoul and Mitri 2016; Hibbing et al. 2010; Hoffmann and Hercus 2000). Bacterial competition involves any mechanism used to harm or exclude another strain or species of bacteria that benefits from the same resources (Grognard et al. 2015; Kawai and Tokeshi 2007). Bacteria compete directly and indirectly. When directly competing, bacteria use metabolites such as antibiotics or other antibiosis molecules secreted via specific competitive secretion systems to inhibit the growth of potential competitors (Hurtado et al. 2011; Morgado and Vicente 2022). When bacteria indirectly compete, they use mechanisms to exploit resources and prevent other microbes from accessing these resources (Coyte and Rakoff‐Nahoum 2019; Ghoul and Mitri 2016). Indirect competition can also result from species that negatively correlate due to varying responses to biotic or abiotic changes, which may improve or reduce fitness within that environment (Kodera et al. 2022).

The SGH predicts that under high stress, bacteria will shift towards facilitative interactions. Facilitative interactions are far less explored in bacteria than competitive interactions (Jorna et al. 2024). Any interaction between two different species resulting in net positive fitness can be considered facilitative. Bacterial facilitative interactions include detoxifying the environment (Hesse et al. 2021) or interactions between species that aid in access to resources such as bacterial translocation by fungi away from stressors or towards nutrient patches (Deveau et al. 2018; Zhang et al. 2018).

In literature, controversies exist between studies of interactions between bacterial community members. These stem from differences observed in laboratory environments such as model communities and co‐culture methods, where negative interactions seem to dominate (Baichman‐Kass et al. 2023; Palmer and Foster 2022), and in natural environments, where positive interactions seem to dominate and can allow species not generally suited for certain environments to thrive (Blasche et al. 2021; Luo et al. 2021; Mandakovic et al. 2018; Xing et al. 2023). This suggests a need for holistic approaches where both laboratory and natural studies are performed to compare variables such as model versus natural community compositions, differences in nutrient composition, substrate type, temperature, and in the previously mentioned biotic factors such as proximities to plant species.

Heavy metals are an excellent abiotic stress to test the SGH in microbes (Naila et al. 2019). Heavy metals are an environmentally relevant abiotic stress with single direct impacts on organisms (Rajapaksha et al. 2004; Singh and Narzary 2021). Other stresses, like salinity and drought, are compound stresses involving multiple stressors. In addition, evidence suggests heavy metals shift bacterial behaviour from competitive to facilitative. For example, in a compost microbiome system, copper shifted inter‐species interactions towards facilitation in which species produced small amounts of metal‐detoxifying siderophores that provided a community benefit within co‐cultures (Hesse et al. 2021).

To better define how heavy metal stress pushes bacteria along a continuum from competitive to facilitative as predicted by the SGH, we focus on the heavy metal selenium (Se) and examine the potential for changes in Se concentration to drive species interactions from competitive to facilitative in bacteria. However, we expect the behaviours and shifts we observe in response to Se will occur for other heavy metals, and thus we reference other heavy metals in our discussion as well. Se is a naturally occurring heavy metal in soils known to be toxic to microbes. It is passively taken up by plants via sulphur (S) transferases due to the similar chemical natures of S and Se (Trippe and Pilon‐Smits 2021). Se naturally occurs in the bedrock of the earth's crust and, through weathering, cycling and runoff processes, occurs naturally in soils, water and in the air (Winkel et al. 2012). It can also act as an anthropogenic pollutant released through uranium and vanadium mining (Chen et al. 2005; Rosenfeld et al. 2018), and as an agricultural supplement (Rayman 2012; White and Broadley 2009). Se acts as an oxidative stress (when in an inorganic form) and creates malformed proteins when incorporated as selenocysteine during translation (Van Hoewyk and Cakir 2017). This causes high protein turnover, an excess of free radicals, and losses in essential physiological functions (Labunskyy et al. 2014). In response to Se stress, bacteria both tolerate and metabolise Se for various cellular processes (Azaizeh et al. 1997; Dowdle and Oremland 1998). These metabolic processes include detoxification and the ability to use Se in respiration as a terminal electron acceptor (Kushwaha et al. 2021). Both Se reduction and oxidation have been confirmed to be microbially driven (Rosenfeld et al. 2017; Schellenger et al. 2015). Thus, Se acts as a stress for bacteria, but there are clear bacterial tolerance mechanisms and facilitative behaviours associated with Se.

Here, we use Se as a sample stressor to address whether the SGH influences species interactions between bacteria with the following questions: (i) What is and how do we assess competition in bacterial microbiomes? (ii) What is and how do we assess facilitation in Se stressed microbiomes? and (iii) How do we tell when interactions shift from competitive to facilitative under Se stress? We also propose hypotheses for future research in this and similar systems.

## 2 Selenium as a Stressor

Stresses, like Se, reduce the area of “unstressed” space bacteria can colonise (Wang et al. 2022; Winkel et al. 2012). Within soils a number of factors determine in what niches or sub‐environment Se may be found, and as Se concentration increases, we expect a greater proportion of soil niches or sub‐environments to contain Se. Soil aggregate size likely influences how Se is distributed in soils, because larger soil particles may retain more Se (Kausch et al. 2012). Additionally, forms of Se (e.g., reduced or oxidised forms) differ in their distribution based on conditions like soil type, geological conditions and microbial species present (Jason et al. 2018; Kausch et al. 2012; Nancharaiah and Lens 2015). In soils, bioavailable forms of Se exist on particles of weathered bedrock that become adsorbed to soil aggregates where bacteria live (Favorito et al. 2021). Thus, bacteria likely experience patches of Se within the soil environment.

Bioavailable Se exists as water‐soluble Se oxyanions like selenate and selenite and can move throughout a system via water flow due to snowfall or rain (Winkel et al. 2015; Yang et al. 2022). Soil runoff from high Se environments can enter aquatic systems and contribute to stress in these systems (Hamilton and Buhl 2005; Yao et al. 2007). Se levels in water are considered high at levels greater than 0.05 mg/L (WHO 2022) for human toxicity, but environmental toxicity for non‐human organisms appears undefined (Vinceti et al. 2013). Like soils, Se in water tends to have heterogeneous distributions as Se levels can differ by water depth, and in the case of streams, by distance from the origin of soil runoff (Cianciolo et al. 2020; Tian et al. 2016). Bioavailable Se in soil and aquatic systems is passively absorbed via sulphur transferases and causes toxicity (Trippe and Pilon‐Smits 2021); however, in water, the species of Se (type of compound) can be changed due to the effects water pH can have on Se redox reactions (Abejón 2022).

Due to the patchy nature of soils, Se may exist in heterogeneous distributions throughout an affected area (Wang et al. 2022). Between soil aggregates, water spaces also provide an environment for bacteria to inhabit in planktonic forms. However, as Se oxyanions flow via water, spaces without Se may be limited. Se oxyanions are soluble and therefore, homogenously distributed. Non‐bioavailable forms of Se, like elemental Se, can be oxidised by weathering or by the metabolic processes of bacteria and then become bioavailable (Sarathchandra and Watkinson 1981; Szeleg et al. 2013). As elemental Se oxidation is a microbial process, soil spaces rich in this form and low in bioavailable forms may be precious in Se‐impacted environments (Dowdle and Oremland 1998).

## 3 What Is and How Do We Measure Competition in Bacterial Microbiomes?

Direct competition between bacteria often involves the release of antibacterial compounds like colicins and bacteriocins which function to prevent the growth of the competing colony (Hurtado et al. 2011; Nakamaru and Iwasa 2000). Competition like this can increase reactive oxygen species (ROS) in response to free radicals promoted by a competitor (Dong et al. 2015). In direct competition, most of the “action” occurs at the interface of the meeting of two colonies where contact‐dependent growth inhibitors lead to the eventual “winning” of one colony (Zhang et al. 2023). An example of a contact‐dependent inhibitor is the Type 6 Secretion system which functions to inject toxins into a competing species (Morgado and Vicente 2022). Disrupting quorum sensing signalling in competing species can also act as competition because it prevents communication between sister cells (Stubbendieck and Straight 2016).

Indirect competition between bacteria often prevents access to resources by other species. Bacteria indirectly competing can use or remove substrates needed by a competitor, or convert that substrate into an unusable form (Ghoul and Mitri 2016). Being the first to access a nutrient patch via advanced motility aids also provides indirect competitive benefits to competing species (An et al. 2006), whereas advantageous enzymes or lipids can utilise substrates before a competitor (Ghoul and Mitri 2016). Similarly, gains or losses in fitness due to biotic and abiotic environmental shifts provide opportunities for species to indirectly compete for resources (Kodera et al. 2022).

Investigating competitive interactions in bacteria is varied, and methodologies are frequently being developed to understand the phenotypic and genotypic roles at play in competitive interactions (Booth et al. 2023; Nguyen‐Le et al. 2023). The Table 1 below provides a brief overview of available methods to measure direct and indirect interactions.

**TABLE 1:** Examples of competitive strategies and how to measure them.

Competitive strategy	Possible measurement	References
*Direct*		
Antibiotics	Co–culture tests with a known antibiotic producing species. Isolating antibiotics, testing on multiple species. Measure ROS production during interactions	Cascales et al. (2007); Roemhild et al. (2022)
Type VI Secretion Systems (T6SS)	Manipulating T6SS expression levels in co‐culture. Manipulating co‐culture partners to determine the breadth of toxins effect	Cascales and Cambillau (2012); Morgado and Vicente 2022)
Quorum Sensing Disruption	Isolating quorum sensing molecules, testing effects on competitors	An et al. (2006)
*Indirect*		
Enzyme Secretion	Measure siderophore production in competition. Measure enzymatic secretions. Measure growth in competitor's supernatant	Harrison et al. (2008); Schmitz et al. (2024); Tabasco et al. (2014)
Motility Advantages	Test if motile bacteria colonise more space. Inhibit flagella genes, measure occupation of space	Gude et al. (2020); Klimenko et al. (2021)

To address the SGH, there are many laboratory approaches for measuring bacterial interactions across solid and liquid substrates for cultured bacteria. The method you choose should be based off a hypothesised expectation to how the interaction happens. For example, in solid spaces, bacteria often “fence off” space by forming colonies using biofilms that prevent access to non‐sister species (Costerton et al. 1999; Drescher et al. 2014). These colonies allow bacteria to perform both exploitative (indirect) and interference (direct) competitive behaviours. Biofilms grow as bacteria divide and as resources are used up, expand, and compete with biofilms of other species (Drescher et al. 2014; Hall‐Stoodley et al. 2004). Biofilms act as barriers that prevent other bacterial species from entering and exploiting the resources of the established colony, and may prevent the growth of competitors (Woo and Ahn 2013). Biofilms can increase their competitive advantage by releasing molecules that prevent the growth of or cause the death of neighbouring colonies (Drescher et al. 2014). In aquatic conditions, exploitative competition would be more frequent as bacteria attempt to utilise free flowing dissolved nutrients as they are available (Hibbing et al. 2010; Nicholson 1954). This limits the access of nutrients to their competitors and further causes growth differences. In model soil communities, methodologies like massively parallel screens could be used to identify soil Se levels that shift interactions towards those net facilitative interactions (Kehe et al. 2019). This method could also be used to identify keystone species which may be driving the positive or negative interactions within those soil communities, which can then be isolated and tested in culture methodologies.

Based on the SGH, at low Se stress we expect bacterial competition to be high in soils. Evidence suggests that bacterial competition is highest in environments where nutritional substrates overlap, and that soil environments tend to generally have more competitive interactions (Machado et al. 2021). This is supported by research showing high nutrient availability increases negative interactions within a soil actinobacteria community (Myers et al. 2012; Yan et al. 2021).

## 4 What Is and How Do We Measure Facilitation in Se Stressed Microbiomes?

Bacterial facilitation takes many forms. Bacteria produce public goods often in the form of extracellular products that can aid the survival and growth of members of the surrounding community (Smith and Schuster 2019). These are usually secondary metabolites such as nutritional excretions, enzymes and signalling compounds which can be used by other species within the community for survival and occur frequently in multi‐species biofilms (Zachar and Boza 2022). Waste removal traits in bacteria are also important and prevent the accumulation of toxic byproducts that stifle community growth by providing additional abiotic stresses (Zachar and Boza 2022). Given this, waste removal may also recycle nutrients within the community and prevent loss of access to these nutrients due to metabolism. Multi‐species or shared biofilms are collaborative bacterial communities that involve co‐operative behaviours and secretions between multiple bacterial species in order to establish and propagate (Joshi et al. 2021), and have evolved mechanisms to prevent “cheaters” from gaining access to their public goods (Smith and Schuster 2019).

Individual traits can also be facilitative outside of shared biofilms. As mentioned before, metal detoxification traits appear to provide a community benefit to non‐tolerant bacterial species (Hesse et al. 2021). This behaviour is sometimes necessary for community survival, as growth inhibitory effects are seen when facilitative partners are not present (Piccardi et al. 2019). Other important microbial traits, like those involved in nutrient cycling, such as carbon sequestration and nitrogen fixation, also provide indirect benefits to communities by increasing access to nutrients (Jorna et al. 2024).

Bacteria may also receive facilitative benefits from fungal community members. “Hyphal migration” (Warmink et al. 2011; Zhang et al. 2018) occurs when bacteria use fungal hyphal networks to travel between sites. These “hyphal highways” are used by bacteria to exploit nutrient patches and could be used to escape stressors, but we know of no studies examining this possibility. Lastly, evidence suggests that obligate commensalisms also exist between bacteria and archaea, which function by providing access to nutrition and waste removal required by both species (Stams and Plugge 2009).

Under high stress, the SGH predicts facilitative behaviours to increase. Mechanisms in bacteria that support facilitative behaviours under high stress environments include heavy metal detoxification mechanisms (Hesse et al. 2021; Piccardi et al. 2019). In soils, as a system becomes loaded with bioavailable Se oxyanions, available spaces of resource‐rich nutrient patches become scarcer, whereas larger regions are now saturated with toxins (Chang et al. 2019). Rising environmental toxicity is likely to lead to species loss unless survival is facilitated. Some species are known to tolerate Se while others can convert Se oxyanions into non‐toxic forms (Eswayah et al. 2016; Nancharaiah and Lens 2015) such as volatile organoselenium compounds released from soils as gases, and elemental Se produced from the reduction of Se oxyanions (Moreno‐Martin et al. 2021). This indirect facilitation can detoxify the environment for non‐Se tolerant bacteria allowing them to persist. This has been shown to occur in aquatic systems (Ivanenko 2018), suggesting this behaviour could also occur in water spaces between soil aggregates.

Bacterial species are capable of Se processing and therefore removing Se and facilitating neighbour species via several pathways. Some selenium‐tolerant bacteria are directly capable of reducing the Se oxyanions, selenite (SeO_3_^2−^) and selenate (SeO_4_^3−^) (Nancharaiah and Lens 2015; Schellenger et al. 2015; Zhang et al. 2019). Reduction can provide metabolic energy for some species of bacteria while detoxifying the soil of these two oxyanions (Eswayah et al. 2016). Depending on the bacterial species, reduction occurs in aerobic, anaerobic or both conditions (Bebien et al. 2001; Klonowska et al. 2005; Li et al. 2014). Selenite and selenate reduction can occur both intracellularly (Bebien et al. 2001; Wang et al. 2018) and extracellularly (Yamada et al. 1997), but the ecological benefits of using either mechanism are not yet understood.

Chemoautotrophic oxidation of elemental selenium can also facilitate neighbour species and occurs in both aquatic and terrestrial ecosystems (Dowdle and Oremland 1998; Ivanenko 2018; Kushwaha et al. 2021) but is less frequently reported than reduction mechanisms. Oxidation mechanisms convert elemental Se back to the bioavailable, inorganic forms of Se (selenite (SeO_3_^2−^) and selenate (SeO_4_^3−^)) (Ivanenko 2018; Sarathchandra and Watkinson 1981) which contributes to the recycling of Se throughout ecosystems (Nancharaiah and Lens 2015). In soil systems, the mechanism of Se oxidation occurs 3–4 times slower than reduction rates (Dowdle and Oremland 1998), but there are factors that can increase the rate of Se oxidation such as enriching bacteria with inorganic forms of C as opposed to glucose (Losi and Frakenberger 1998).

## 5 When Could the Shift Between Competition and Facilitation Happen?

The SGH predicts that as a given stress reaches a threshold, species interactions switch from net competitive to net facilitative interactions. This suggests a concentration or intensity dependent mechanism that allows bacterial co‐existence under stress and that likely varies with each stress. Net positive interactions do not prevent competition from occurring, and competition remains an essential factor controlling populations (Cadotte 2023; He et al. 2013). There are two complementary approaches for assessing when and where the shift between competition and facilitation occurs: laboratory co‐cultures and field collections.

Laboratory co‐culture experiments reveal co‐operating/competing dynamics of interactions and allow us to determine where a “switch” from net competitive to net facilitative interactions occurs. Co‐culture experiments can be modified from existing competition experiments (Table 1) to focus on growth aid instead of growth inhibition. Manipulating stress levels experimentally allows us to pinpoint where species traits become beneficial to a community, as well as understand which species in a community provide these benefits (Di Martino et al. 2024). In co‐culture experiments, in conjunction with massively parallel screens, we can also manipulate community size, trait distributions, and assess which community members are lost and maintained throughout the growth period (Castledine et al. 2024; Kehe et al. 2019). Understanding these processes allows us to transition to soil systems where variations in stress are often multifactored.

In the field, we can use laboratory assays, metagenomics and metatranscriptomics to selectively target certain traits and community members to determine if their presence within the community has shifted as stress increases. When facilitation occurs, we hypothesise a species that could be lost due to stress may be rescued by neighbouring species hosting traits that reduce stress in their shared environment. Under field conditions, we could observe a vulnerable species occurring with a facilitative species more often than expected under high stress as opposed to low stress. Microbial communities are dynamic and shift constantly in response to the environment and other microbes (Hammarlund and Harcombe 2019). Thus, we recommend multiple sampling times and points to determine when stress drives the switch in prevalence of competition and facilitation.

## 6 Bacterial Community Species Richness and the SGH

The ability of bacteria to move along the competitive‐facilitative continuum as predicted by the SGH is due to the variability of metabolic functions and stress tolerance within a bacterial community. Does this suggest that a highly diverse population is required for the SGH to occur? Could it be possible that species diversity and, by extension, genetic diversity allow for community stress resilience during periods of environmental stress? Most studies regarding the SGH focus on small numbers of interacting species (usually two) due to the difficulty in assessing multi‐way interactions. But bacterial communities are substantially larger and involve potentially more interactions than other systems. For example, 1 g of soil is estimated to contain 10^8^–10^10^ bacterial cells (Raynaud and Nunan 2014), which opens the potential for millions of interactions.

Carrying capacity is the maximum population size systems can support before resource exhaustion and population decline (Brown et al. 2004; Lidicker 1962). In competition experiments, populations often grow rapidly, and population size is only limited by the resources present within their environment. However, in multi‐species communities, the carrying capacity of a species is smaller as competing species can acquire resources as well. However, the effects of abiotic stress and environmental conditions on carrying capacity are severely understudied in bacteria (Foley et al. 2024).

With the emergence of bioremediation applications that use bacteria and bacterial cocktails to help remediate soils or systems, the ecological effects that stress would have on these interactions are vital. Using the SGH and model communities to assess the effectiveness of already stressed biomes allows for more controlled attempts at remediation, as the SGH could be used to determine multifactor stress responses in model remediation communities. In lab environments, methodologies to study these interactions are relatively new (Sprouffske and Wagner 2016; Temkin et al. 2019), but offer insightful knowledge into bacterial community size, their interactions, and their outcomes under stress.

## 7 Conclusion

Overall, bacterial communities provide an exciting avenue to study the SGH. We can apply the SGH to multifactor stresses, natural communities and utilise our findings in soil remediation and conservation. Additionally, understanding how bacterial communities respond to disturbances helps us understand how biogeochemical and nutrient cycling functions are conserved in periods of stress.

Exploring bacterial mechanisms of facilitation and stress tolerance also allows us to determine the ecological relevance of poorly defined traits. For example, traits focused on absorbing toxins and detoxifying them through metabolism benefit neighbours who may struggle when the toxin is present. Likewise, converting a stressor to a substrate that can be processed by a neighbour also provides essential services to the community. We may also be able to uncover traits that allow for co‐existence that may not directly relate to abiotic stress removal.

As microbiome research progresses and incorporates metagenomics, we can explore how the distribution of genes in a community relates to traits and functions in a community. Understanding how traits allow for species co‐existence is integral to understanding which genes in a community are important for community composition and stability. Future research will address how microbial ecological traits become prevalent, change, or are lost under stress.

## Author Contributions

**Kristian J. Harris:** conceptualization, investigation, writing – original draft, writing – review and editing, methodology. **Alison E. Bennett:** conceptualization, writing – review and editing, supervision, resources.

## Conflicts of Interest

The authors declare no conflicts of interest.

## Data Availability

Data sharing not applicable to this article as no datasets were generated or analysed during the current study.
